# 4-(2*H*-Tetra­zol-5-yl)pyridinium hydrogen sulfate

**DOI:** 10.1107/S1600536809032851

**Published:** 2009-09-09

**Authors:** Bo Wang

**Affiliations:** a Ordered Matter Science Research Center, College of Chemistry and Chemical Engineering, Southeast University, Nanjing 210096, People’s Republic of China

## Abstract

In the cation of the title compound, C_6_H_6_N_5_^+^·HSO_4_^−^, the pyridine and tetra­zole rings are close to being co-planar [dihedral angle = 3.98 (7)°]. In the crystal, the ions are linked by O—H⋯O, N—H⋯O and N—H⋯(O,O) hydrogen bonds, resulting in chains.

## Related literature

Tetrazoles are excellent ligands for the construction of metal-organic frameworks because of their various coordination modes, see: Fu *et al.* (2008 ▶); Wang *et al.* (2005 ▶). For the applications of metal-organic coordination compounds, see: Fu *et al.* (2007 ▶); Huang *et al.* (1999 ▶); Liu *et al.* (1999 ▶); Xie *et al.* (2003 ▶); Zhang *et al.* (2001 ▶); Zhang *et al.* (2000 ▶). 
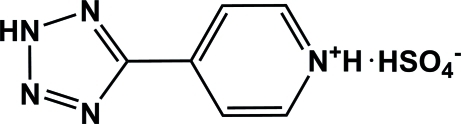


## Experimental

### 

#### Crystal data

C_6_H_6_N_5_^+^·HSO_4_^−^
*M* *_r_* = 245.23
Triclinic, 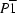

*a* = 6.6515 (13) Å
*b* = 7.5507 (15) Å
*c* = 10.072 (2) Å
α = 77.72 (3)°
β = 76.88 (3)°
γ = 79.71 (3)°
*V* = 476.84 (16) Å^3^
*Z* = 2
Mo *K*α radiation
μ = 0.35 mm^−1^
*T* = 298 K
0.30 × 0.25 × 0.20 mm

#### Data collection

Rigaku Mercury2 diffractometer
Absorption correction: multi-scan (*CrystalClear*; Rigaku, 2005 ▶) *T*_min_ = 0.910, *T*_max_ = 1.000 (expected range = 0.848–0.932)
4917 measured reflections
2175 independent reflections
1961 reflections with *I* > 2σ(*I*)
*R*_int_ = 0.055

#### Refinement

*R*[*F* ^2^ > 2σ(*F* ^2^)] = 0.050
*wR*(*F* ^2^) = 0.134
*S* = 1.07
2175 reflections
146 parameters
H-atom parameters constrained
Δρ_max_ = 0.34 e Å^−3^
Δρ_min_ = −0.41 e Å^−3^

### 

Data collection: *CrystalClear* (Rigaku, 2005 ▶); cell refinement: *CrystalClear*; data reduction: *CrystalClear*; program(s) used to solve structure: *SHELXS97* (Sheldrick, 2008 ▶); program(s) used to refine structure: *SHELXL97* (Sheldrick, 2008 ▶); molecular graphics: *SHELXTL* (Sheldrick, 2008 ▶); software used to prepare material for publication: *SHELXTL*.

## Supplementary Material

Crystal structure: contains datablocks I, global. DOI: 10.1107/S1600536809032851/bx2220sup1.cif

Structure factors: contains datablocks I. DOI: 10.1107/S1600536809032851/bx2220Isup2.hkl

Additional supplementary materials:  crystallographic information; 3D view; checkCIF report

## Figures and Tables

**Table 1:** Hydrogen-bond geometry (Å, °)

*D*—H⋯*A*	*D*—H	H⋯*A*	*D*⋯*A*	*D*—H⋯*A*
O1—H1*B*⋯O2^i^	0.82	1.90	2.694 (2)	163
N1—H1*A*⋯O4^ii^	0.86	1.91	2.736 (2)	159
N4—H4*A*⋯O2^iii^	0.86	2.57	3.033 (3)	115
N4—H4*A*⋯O3	0.86	1.97	2.741 (2)	150

Symmetry codes: (i) 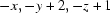; (ii) 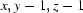; (iii) 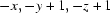.

**Fig. 1.**
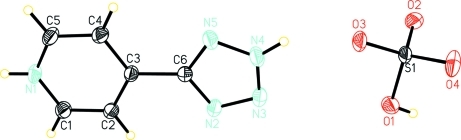
A view of the title compound with the atomic numbering scheme. Displacement ellipsoids were drawn at the 30% probability level.

**Fig. 2.**
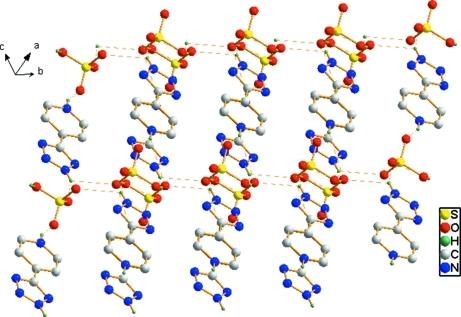
The crystal packing of the title compound showing the two dimensionnal hydrogen bondings network (dashed line). Hydrogen atoms not involved in hydrogen bonding have been omitted for clarity.

## supplementary crystallographic information

### Comment

The construction of metal-organic coordination compounds has attracted much attention owing to the potential functions, such as permittivity, fluorescence, magnetism and optical properties. (Fu *et al.*, 2007; Huang *et al.*, 1999; Liu *et al.*, 1999; Xie *et al.*, 2003; Zhang *et al.*,2001; Zhang *et al.*,2000) Tetrazole compounds are a class of excellent ligands for the construction of novel metal-organic frameworks, because of its various coordination modes. (Wang, *et al.* 2005; Fu *et al.*, 2008). We report here the crystal and molecular structure of the title compound, 4-(2*H*-tetrazol-5-yl)pyridinium bisulfate), (I) , Fig.1, The pyridine N atoms are protonated. The pyridine and tetrazole rings are nearly coplanar and only twisted from each other by a dihedral angle of 3.98 (7) °. The geometric parameters of the tetrazole rings are comparable to those in related molecules (Wang, *et al.* 2005; Fu *et al.*, 2008).The crystal packing is stabilized by coulombic forces , one O—H···O and  three N—H···O hydrogen bonds (Table 1 and Fig.2).

### Experimental

Isonicotinonitrile (30 mmol), NaN _3_ (45 mmol), NH_4_Cl (33 mmol) and DMF (50 ml) were added in a flask under nitrogen atmosphere and the mixture stirred at 110°C for 20 h. The resulting solution was then poured into ice-water (100 ml), and a white solid was obtained after adding HCl (6 *M*) still pH=6. The precipitate was filtered and washed with distilled water. Colourless block-shaped crystals suitable for X-ray analysis were obtained from the crude product by slow evaporation of an ethanol/H_2_SO_4_ (50:1 *v*/*v*) solution.

### Refinement

All H atoms attached to C, O and N atoms were fixed geometrically and treated as riding with C–H = 0.93 Å, O–H = 0.82 Å and N–H = 0.86 Å with *U*_iso_(H) =1.2Ueq(C or N) and *U*_iso_(H) =1.5Ueq(O).

### Crystal data

**Table:**

C_6_H_6_N_5_^+^·HSO_4_^−^	*Z* = 2
*M**_r_* = 245.23	*F*(000) = 252
Triclinic, *P*1	*D*_x_ = 1.708 Mg m^−^^3^
Hall symbol: -P 1	Mo *K*α radiation, λ = 0.71073 Å
*a* = 6.6515 (13) Å	Cell parameters from 1961 reflections
*b* = 7.5507 (15) Å	θ = 3.2–27.5°
*c* = 10.072 (2) Å	µ = 0.35 mm^−^^1^
α = 77.72 (3)°	*T* = 298 K
β = 76.88 (3)°	Block, colorless
γ = 79.71 (3)°	0.30 × 0.25 × 0.20 mm
*V* = 476.84 (16) Å^3^	

### Data collection

**Table:**

Rigaku Mercury2 diffractometer	2175 independent reflections
Radiation source: fine-focus sealed tube	1961 reflections with *I* > 2σ(*I*)
graphite	*R*_int_ = 0.055
Detector resolution: 13.6612 pixels mm^-1^	θ_max_ = 27.5°, θ_min_ = 3.2°
CCD profile fitting scans	*h* = −8→8
Absorption correction: multi-scan (*CrystalClear*; Rigaku, 2005)	*k* = −9→9
*T*_min_ = 0.910, *T*_max_ = 1.000	*l* = −13→13
4917 measured reflections	

### Refinement

**Table:**

Refinement on *F*^2^	Secondary atom site location: difference Fourier map
Least-squares matrix: full	Hydrogen site location: inferred from neighbouring sites
*R*[*F*^2^ > 2σ(*F*^2^)] = 0.050	H-atom parameters constrained
*wR*(*F*^2^) = 0.134	*w* = 1/[σ^2^(*F*_o_^2^) + (0.0597*P*)^2^ + 0.1736*P*] where *P* = (*F*_o_^2^ + 2*F*_c_^2^)/3
*S* = 1.07	(Δ/σ)_max_ < 0.001
2175 reflections	Δρ_max_ = 0.34 e Å^−^^3^
146 parameters	Δρ_min_ = −0.40 e Å^−^^3^
0 restraints	Extinction correction: *SHELXL97* (Sheldrick, 2008), Fc^*^=kFc[1+0.001xFc^2^λ^3^/sin(2θ)]^-1/4^
Primary atom site location: structure-invariant direct methods	Extinction coefficient: 0.137 (14)

### Special details

**Table:**

Geometry. All e.s.d.'s (except the e.s.d. in the dihedral angle between two l.s. planes) are estimated using the full covariance matrix. The cell e.s.d.'s are taken into account individually in the estimation of e.s.d.'s in distances, angles and torsion angles; correlations between e.s.d.'s in cell parameters are only used when they are defined by crystal symmetry. An approximate (isotropic) treatment of cell e.s.d.'s is used for estimating e.s.d.'s involving l.s. planes.
Refinement. Refinement of *F*^2^ against ALL reflections. The weighted *R*-factor *wR* and goodness of fit *S* are based on *F*^2^, conventional *R*-factors *R* are based on *F*, with *F* set to zero for negative *F*^2^. The threshold expression of *F*^2^ > σ(*F*^2^) is used only for calculating *R*-factors(gt) *etc*. and is not relevant to the choice of reflections for refinement. *R*-factors based on *F*^2^ are statistically about twice as large as those based on *F*, and *R*- factors based on ALL data will be even larger.

### Fractional atomic coordinates and isotropic or equivalent isotropic displacement parameters (Å^2^)

**Table:**

	*x*	*y*	*z*	*U*_iso_*/*U*_eq_	
S1	0.17610 (8)	0.76548 (6)	0.48163 (5)	0.0345 (2)	
O2	−0.0454 (3)	0.7896 (2)	0.54236 (17)	0.0469 (4)	
O1	0.2068 (3)	0.9101 (2)	0.34498 (15)	0.0469 (4)	
H1B	0.1631	1.0128	0.3627	0.070*	
C6	0.2581 (3)	0.3789 (3)	0.0215 (2)	0.0321 (4)	
N2	0.2528 (3)	0.5615 (2)	−0.00724 (18)	0.0394 (4)	
O3	0.2479 (3)	0.5928 (2)	0.43634 (16)	0.0445 (4)	
N1	0.2800 (3)	0.0732 (2)	−0.28273 (19)	0.0397 (4)	
H1A	0.2824	0.0112	−0.3456	0.048*	
O4	0.3022 (3)	0.8031 (2)	0.56937 (19)	0.0537 (5)	
N3	0.2423 (3)	0.6128 (3)	0.1106 (2)	0.0438 (5)	
N4	0.2414 (3)	0.4632 (3)	0.20321 (19)	0.0447 (5)	
H4A	0.2349	0.4630	0.2895	0.054*	
N5	0.2511 (3)	0.3125 (3)	0.15493 (18)	0.0444 (5)	
C2	0.2855 (3)	0.3527 (3)	−0.2218 (2)	0.0363 (5)	
H2	0.2935	0.4774	−0.2472	0.044*	
C3	0.2684 (3)	0.2687 (3)	−0.0841 (2)	0.0305 (4)	
C4	0.2597 (3)	0.0814 (3)	−0.0490 (2)	0.0374 (5)	
H4	0.2498	0.0217	0.0426	0.045*	
C5	0.2661 (4)	−0.0136 (3)	−0.1518 (2)	0.0415 (5)	
H5	0.2606	−0.1389	−0.1303	0.050*	
C1	0.2904 (4)	0.2516 (3)	−0.3202 (2)	0.0395 (5)	
H1	0.3010	0.3072	−0.4128	0.047*	

### Atomic displacement parameters (Å^2^)

**Table:**

	*U*^11^	*U*^22^	*U*^33^	*U*^12^	*U*^13^	*U*^23^
S1	0.0519 (4)	0.0259 (3)	0.0276 (3)	−0.0071 (2)	−0.0060 (2)	−0.00971 (19)
O2	0.0546 (10)	0.0378 (8)	0.0457 (9)	−0.0136 (7)	0.0045 (7)	−0.0116 (7)
O1	0.0696 (11)	0.0312 (8)	0.0346 (8)	−0.0076 (7)	0.0002 (7)	−0.0046 (6)
C6	0.0385 (10)	0.0295 (9)	0.0286 (9)	−0.0064 (8)	−0.0048 (7)	−0.0064 (7)
N2	0.0534 (11)	0.0322 (9)	0.0353 (9)	−0.0087 (8)	−0.0078 (8)	−0.0105 (7)
O3	0.0711 (11)	0.0288 (8)	0.0365 (8)	−0.0053 (7)	−0.0095 (7)	−0.0146 (6)
N1	0.0495 (10)	0.0379 (10)	0.0384 (10)	−0.0063 (8)	−0.0112 (8)	−0.0181 (8)
O4	0.0735 (12)	0.0439 (9)	0.0561 (10)	−0.0003 (8)	−0.0281 (9)	−0.0259 (8)
N3	0.0573 (11)	0.0383 (10)	0.0399 (10)	−0.0082 (8)	−0.0081 (8)	−0.0160 (8)
N4	0.0634 (13)	0.0434 (10)	0.0290 (9)	−0.0042 (9)	−0.0081 (8)	−0.0141 (7)
N5	0.0654 (13)	0.0396 (10)	0.0284 (9)	−0.0074 (9)	−0.0058 (8)	−0.0099 (7)
C2	0.0487 (12)	0.0312 (10)	0.0309 (10)	−0.0111 (9)	−0.0078 (8)	−0.0052 (8)
C3	0.0349 (9)	0.0283 (9)	0.0304 (9)	−0.0050 (7)	−0.0076 (7)	−0.0082 (7)
C4	0.0495 (12)	0.0300 (10)	0.0335 (10)	−0.0082 (9)	−0.0098 (8)	−0.0032 (8)
C5	0.0549 (13)	0.0261 (10)	0.0462 (12)	−0.0066 (9)	−0.0130 (10)	−0.0080 (8)
C1	0.0524 (12)	0.0380 (11)	0.0305 (10)	−0.0098 (9)	−0.0101 (9)	−0.0062 (8)

### Geometric parameters (Å, °)

**Table:**

S1—O3	1.4378 (15)	N3—N4	1.302 (3)
S1—O4	1.4469 (17)	N4—N5	1.315 (3)
S1—O2	1.4564 (17)	N4—H4A	0.8600
S1—O1	1.5623 (16)	C2—C1	1.365 (3)
O1—H1B	0.8200	C2—C3	1.385 (3)
C6—N5	1.324 (3)	C2—H2	0.9300
C6—N2	1.343 (3)	C3—C4	1.392 (3)
C6—C3	1.466 (3)	C4—C5	1.369 (3)
N2—N3	1.309 (2)	C4—H4	0.9300
N1—C1	1.329 (3)	C5—H5	0.9300
N1—C5	1.332 (3)	C1—H1	0.9300
N1—H1A	0.8600		
			
O3—S1—O4	112.84 (10)	N5—N4—H4A	122.5
O3—S1—O2	113.35 (10)	N4—N5—C6	100.98 (18)
O4—S1—O2	112.29 (11)	C1—C2—C3	119.79 (19)
O3—S1—O1	104.09 (9)	C1—C2—H2	120.1
O4—S1—O1	106.97 (11)	C3—C2—H2	120.1
O2—S1—O1	106.53 (10)	C2—C3—C4	119.02 (18)
S1—O1—H1B	109.5	C2—C3—C6	119.51 (18)
N5—C6—N2	112.09 (18)	C4—C3—C6	121.47 (18)
N5—C6—C3	124.79 (18)	C5—C4—C3	118.83 (19)
N2—C6—C3	123.12 (18)	C5—C4—H4	120.6
N3—N2—C6	106.28 (18)	C3—C4—H4	120.6
C1—N1—C5	122.79 (18)	N1—C5—C4	120.08 (19)
C1—N1—H1A	118.6	N1—C5—H5	120.0
C5—N1—H1A	118.6	C4—C5—H5	120.0
N4—N3—N2	105.63 (17)	N1—C1—C2	119.5 (2)
N3—N4—N5	115.02 (17)	N1—C1—H1	120.3
N3—N4—H4A	122.5	C2—C1—H1	120.3
			
C6—N2—N3—N4	−0.1 (2)	C3—C4—C5—N1	−0.1 (3)
N2—N3—N4—N5	0.1 (3)	N4—N5—C6—N2	0.0 (2)
N3—N4—N5—C6	−0.1 (3)	N4—N5—C6—C3	−179.4 (2)
C5—N1—C1—C2	−0.4 (3)	N3—N2—C6—N5	0.0 (2)
N1—C1—C2—C3	−0.4 (3)	N3—N2—C6—C3	179.51 (19)
C1—C2—C3—C4	0.9 (3)	C2—C3—C6—N5	−176.8 (2)
C1—C2—C3—C6	−178.5 (2)	C4—C3—C6—N5	3.8 (3)
C2—C3—C4—C5	−0.7 (3)	C2—C3—C6—N2	3.7 (3)
C6—C3—C4—C5	178.7 (2)	C4—C3—C6—N2	−175.68 (19)
C1—N1—C5—C4	0.7 (3)		

### Hydrogen-bond geometry (Å, °)

**Table:**

*D*—H···*A*	*D*—H	H···*A*	*D*···*A*	*D*—H···*A*
O1—H1B···O2^i^	0.82	1.90	2.694 (2)	163
N1—H1A···O4^ii^	0.86	1.91	2.736 (2)	159
N4—H4A···O2^iii^	0.86	2.57	3.033 (3)	115
N4—H4A···O3	0.86	1.97	2.741 (2)	150

Symmetry codes: (i) −*x*, −*y*+2, −*z*+1; (ii) *x*, *y*−1, *z*−1; (iii) −*x*, −*y*+1, −*z*+1.
